# Cell Membrane-Coated Nanoparticles for Dental, Oral, and Craniofacial Diseases

**DOI:** 10.34133/research.0478

**Published:** 2024-09-18

**Authors:** Kang-Ning Wang, Zi-Zhan Li, Kan Zhou, Bing Liu, Lang Rao, Lin-Lin Bu

**Affiliations:** ^1^ State Key Laboratory of Oral & Maxillofacial Reconstruction and Regeneration, Key Laboratory of Oral Biomedicine Ministry of Education, Hubei Key Laboratory of Stomatology, School & Hospital of Stomatology, Wuhan University, Wuhan 430079, China.; ^2^ Department of Oral & Maxillofacial—Head Neck Oncology, School & Hospital of Stomatology, Wuhan University, Wuhan 430079, China.; ^3^ Institute of Biomedical Health Technology and Engineering, Shenzhen Bay Laboratory, Shenzhen 518132, China.

## Abstract

Dental, oral, and craniofacial diseases can substantially impact the quality of human life, thereby posing a serious public health concern. Although conventional therapies such as surgery have solved these problems largely, the prognosis of patients is not always satisfactory. Cell membrane-coated nanoparticles (CMCNPs) carry nanodrugs with the help of natural cell membranes, therefore utilizing their remarkable ability to interface and interact with their surrounding environment. These nanoparticles have demonstrated substantial advantages in drug targeting, prolonging blood circulation time, penetrating biofilms, and immune escape. With the assistance of CMCNPs, the therapeutic effects of dental, oral, and craniofacial diseases can reach a higher level. CMCNPs have been applied for dental, oral, and craniofacial diseases for various conditions such as head and neck cancer, periodontal disease, and oral biosignal detection. For the therapies of head and neck cancer, CMCNPs have been widely utilized as a tool of chemotherapy, phototherapy, and immunotherapy, while yet to be exploited in imaging technique. In the end, we summarized the challenges and prospectives of CMCNPs for dental, oral, and craniofacial diseases: large-scale production with uniform standards and high quantity, extensive application directions in dental, oral, and craniofacial regions (implant, endodontics), and the promotion of its clinical application.

## Introduction

Dental, oral, and craniofacial diseases have emerged as a pressing public health concern, exhibiting alarmingly high incidence rates and posing serious threats to human longevity and quality of life [1]. Head and neck cancer (HNC) ranks as the sixth most common type of malignancy worldwide, with a dismal 5-year survival rate hovering around 50% [2]. Periodontal disease, notably periodontitis, is a prevalent oral condition affecting a large segment of the population. Its high prevalence can culminate in edentulousness and impaired chewing, substantially compromising quality of life, particularly among the elderly [3]. From 1990 to 2010, the burden of periodontal diseases increased by 57.3% [4]. It is indisputable that over half of the population suffers from periodontitis [5]. In 2019, there were 1.09 billion people affected by periodontitis globally [6]. Other diseases like endodontic diseases are common in daily life. When caries invade nerves, Intensive pain will seriously reduce the quality of life of patients. The prevalence of apical periodontitis in Europe is as high as 34 to 61% of individuals and increases with age [7,8]. As the entrance of food intake, oral and dental diseases are important to human nutrition and they are closely related to systemic diseases such as diabetes and atherosclerosis [9,10]. The ineffectiveness of current treatments for dental, oral, and craniofacial diseases, which deeply disrupt life’s facets, necessitates urgent exploration of innovative therapies.

Nanoparticles (NPs) are particularly effective in drug delivery system (DDS), which can achieve targeted drug delivery and local immunomodulation [11]. Encapsulating medicine within NPs not only improves its stability and solubility but also facilitates transmembrane transport and prolongs the cycle time [12,13]. The integration of NP-based DDS with imaging technology can achieve visualization of drug delivery [14]. However, conventional NP-based DDS has fallen in some aspects. Systemic administration is usually carried out with the aim of covering the lesion as much as possible and not being limited to the known and accessible lesion. During systemic circulation, NP-based DDS is easily blocked by blood flow, coronas, and other factors, and one of the big obstacles is being recognized and cleared by the cells of the mononuclear phagocyte system (MPS) or reticuloendothelial system [12]. Swallowed by MPS, NPs will accumulate in the liver and spleen, potentially triggering immune responses that can lead to inflammation and tissue damage [15]. In the past years, researchers have been exploring how to prevent NPs from being recognized and cleared by the immune system. Many nanocarrier materials now include polyethylene glycol (PEG) as a “stealth coating”, which can prevent aggregation, opsonization, and phagocytosis, thus prolonging the circulation time [16]. Doxil is the first Food and Drug Administration (FDA)-approved nanodrug coated with PEG [17]. While Doxil can achieve a better circulation time than other NPs, with a drug half-life of 72 h and a circulation half-life of 36 h [16], the statistics are far inferior to cell membrane-coated nanoparticles (CMCNPs) (the circulation time of CMCNPs can be up to several days) [18]. Although Doxil can escape the phagocytosis of the MPS, how to realize internalization to be captured by targeted cells is a problem [19]. Besides, this carrier will cause acquired immunity. Once the body has been exposed to PEG, the body can produce PEG antibodies against it, leading to the rapid removal of NPs. As the service time extends, the therapeutic effect gradually deteriorates. Doxil depends on the enhanced permeability and retention (EPR) effect to accumulate in the tumor-vascularized area, so this carrier frequently lacks the necessary active targeting capabilities [17]. Targeted modification of traditional NPs requires a lot of investment of resources and imposes a heavy economic burden on patients [20].

To overcome these challenges, researchers focus on developing bionic nanodelivery systems. CMCNPs have demonstrated remarkable biocompatibility, drawing inspiration from the top-down biomimetic approach of red blood cell membrane (RBCM)-derived vesicles [21]. CMCNPs are core NPs coated with a naturally derived cellular membrane, which can form shell–core nanostructures similar to cells [22]. This bionic NP exhibits high concealment and can escape the clearance of the immune system, prolonging the half-life period of the drug in vivo. The targeting trait of CMCNPs is beneficial to lower the toxicity of drugs and overcome the scouring of drugs in the oral liquid environment. CMCNPs can decrease the side effects that chemotherapy induced like weight loss and hemolysis. This can assist the postoperative management of HNC, eliminate residual lesions, and inhibit metastasis. Furthermore, CMCNPs can interact more effectively with biological substrates or biointerfacing, which is beneficial to specifically act on the targeted site [23,24]. It is very difficult to artificially simulate the complex interaction biofilm in the dielectric conductor. Still, CMCNPs offer a unique combination of biomaterial functionality and engineering material adaptability [25]. This may pave a new path for simplifying the development project of the nanocarrier platform. Currently, various membrane types have been explored, including leukocyte membranes [26], erythrocyte membranes [27], platelet (PLT) membranes [28], cancer cell membranes (CCMS) [29], and bacterial membranes [30]. Researchers have used gingival interdental papilla mesenchymal stromal cells to deliver paclitaxel (PTX) for the inhibition of pancreatic cancer cells [31]. CMCNPs are additionally employed for the efficient synergy of antimicrobial activity and immunomodulation in the treatment of periodontitis, as well as head and neck squamous cell carcinoma (HNSCC) [32,33]. CMCNPs face hurdles in clinical translation due to challenges in large-scale, standardized production, escalating costs, and uncertain efficacy [34]. Moreover, its application in these areas is constrained by limited scope and a lack of clinical trials. Ethical considerations in cellular applications also remain pertinent.

In this review, we provided an overview of CMCNPs for drug delivery in the dental, oral, and craniofacial regions, highlighting both the benefits and limitations of CMCNPs applied for periodontitis and HNC. Notably, these CMCNPs have improved drug efficiency substantially. Additionally, we delve into the materials and production processes involved in the fabrication of CMCNPs. Finally, we summarize the current challenges and future prospects of CMCNPs in dental, oral, and craniofacial applications, providing a thorough understanding of their potential and limitations.

## The Characteristics of CMCNPs Applied in Dental, Oral, and Craniofacial Diseases

### Cell membrane applied in dental, oral, and craniofacial diseases

The properties of CMCNPs differ with the source of the cell membrane, which can be leveraged for dental, oral, and craniofacial applications, and they can be summarized into biocompatibility, targeting, tropism, long circulation half-life, and immune activity.

The contents of erythrocytes are mainly hemoglobin, lacking organelles, which is more conducive to the purification and extraction of cell membranes containing high-density self-markers [23]. The circulation half-life of NPs coated with natural erythrocyte membranes is about twice as long as that of NPs coated with PEG, because of the immunosuppressant protein CD47 on the erythrocyte membrane that inhibits the uptake of macrophages [21,35]. The delay of circulation half-life is beneficial to the accumulation of drugs in the affected area. Similar to erythrocytes, PLTs can achieve invisibility mediated by CD47 protein on the membrane, which can block the uptake of macrophages [36]. The glycoprotein receptor of collagen membrane on PLT membrane can bind with subendothelial components (human type IV collagen), therefore mediating inflammation and wound treatment [37,38].

Immune cells like macrophages have been widely applied in the CMCNP technique for the dental, oral, and craniofacial realm. There is a complex biological relationship between immune cells and tumors, and the tumor prognosis of high-level infiltration by different immune cells may be completely opposite [39]. Macrophage membranes can avoid uptake by the MPS and preferentially bind to inflamed endothelial cells, which is beneficial for NPs to specifically target tumors [26]. Additionally, macrophage membrane-coated NPs have been certified with good biocompatibility in vivo [40]. It is reported that macrophage membranes can imitate the proton sponge effect, which can solve the adverse effect of cell membranes on drug release [41]. After targeted delivery of drugs to tumor microenvironment (TME) rich in H ions, it will induce electrolytes and water to flood into CMCNPs, thus gradually releasing encapsulated drugs in response to pH differences. Cancer cells exhibit the characteristic of isotype binding, thereby enabling NPs coated with these cells to demonstrate substantially enhanced homologous targeting capabilities [42]. The abundant antigens present in CCMs can stimulate the immune system, offering novel insights and avenues for developing innovative tumor-targeting nanovaccines [43].

The receptors on mesenchymal stem cells (MSCs) can combine with vascular endothelial growth factor (VEGF) and PLT-derived growth factor, attach to vascular endothelial cells, and pass through the endodermis of tumor blood vessels, thus achieving the effect of responding to inflammatory factors and targeting tumor tissues [34]. This special cell can achieve self-renewal and differentiation in vitro, so it can be prepared in large quantities to obtain a large number of MSC membranes that endow NPs with high biocompatibility. In addition, exosomes have been widely utilized to deliver drugs for their remarkable capability in transporting copious biological cargos and their intricate entanglement in a myriad of cellular functions [44]. Bacterial extracellular vesicle (bEV) has become a carrier of drug delivery because it can be obtained in large quantities. BEV carries a large number of bacterial antigens and can be swallowed by antigen-presenting cells (APCs), which makes it specific as an adjuvant for vaccines [30]. This trait provides a potential application of bEV in periodontitis bacteria resistances. In addition, bEV can secrete cytokines to induce immune response to damage tumors and their ease of surface modulation can ensure that the response is controlled. Despite its immunogenicity, bEV is prone to elimination within the bloodstream, posing a challenge for sustained efficacy [45]. Furthermore, advancements in both its targeting specificity and drug-loading capacity are necessary to optimize its therapeutic potential.

### Engineering technology for CMCNPs

The deficiency of cell membrane performance can be addressed by engineering materials. To enhance the properties of CMCNPs, engineering functional modifications has been developed, encompassing physical, chemical, and genetic engineering techniques [46].

Lipid insertion and membrane hybridization are common physical engineering techniques. Lipid insertion, utilizing the fluidity of the cell membrane, can partially insert the ligand–linker–lipid conjugate into the lipid bilayer of the membrane to form a physical attachment, which can increase the targeting of DDSs and accumulate drugs at the lesion site on the basis of retaining the intact natural membrane [47,48]. Inserting sensitive liposomes with specific responses into cell membranes can also accelerate the release of drugs in special sites [49,50]. Membrane hybridization entails the fusion of membranes originating from diverse sources, synergistically performing various complex tasks in biologically related environments, through techniques such as ultrasonic treatment and coextrusion with porous membranes [46,51]. Taking bEV, for instance, is an effective method to solve the shortage of targeting and drug-loading performance. The eukaryotic–prokaryotic vesicle assembled by combining bEV with the tumor cell membrane and vesicle integrates various tumor-related antigens, which can achieve 10 times the tumor accumulation quantity of unmodified bEV [52]. This technique has been utilized in HNC therapy, for the multiple targets of hybrid membranes and combinative functions [53,54].

Chemical conjugation indicates modifying various functional groups (e.g., primary amines, carboxylic acids, and free thiols from membrane-associated proteins and polysaccharides) on cell membrane surface molecules by coupling reaction, therefore endowing CMCNPs with accessory functions, offering high efficiency, selectivity, and stability compared with the physical modification methods [55]. For example, the construction of cyanuric chloride-coupled methoxy(polyethylene glycol) (mPEG) on the surface of the cell membrane by nucleophilic substitution reaction can prevent the antigen-presenting effect and reduce the phagocytosis of DDSs by monocytes [56].

Another effective way to modify cell membranes is gene engineering, which transduces the gene to be expressed into the selected cells by virus, then determines the cells expressing target proteins by immunofluorescence and flow cytometry, and extracts the cell membranes [57,58]. Compared with human cell membranes, bacterial cell membranes are easier to modify by genetic engineering, so antigens on the membrane can be designed as needed [59].

### Advantages of CMCNPs applied in the dental, oral, and craniofacial region

**Fig. 1.**
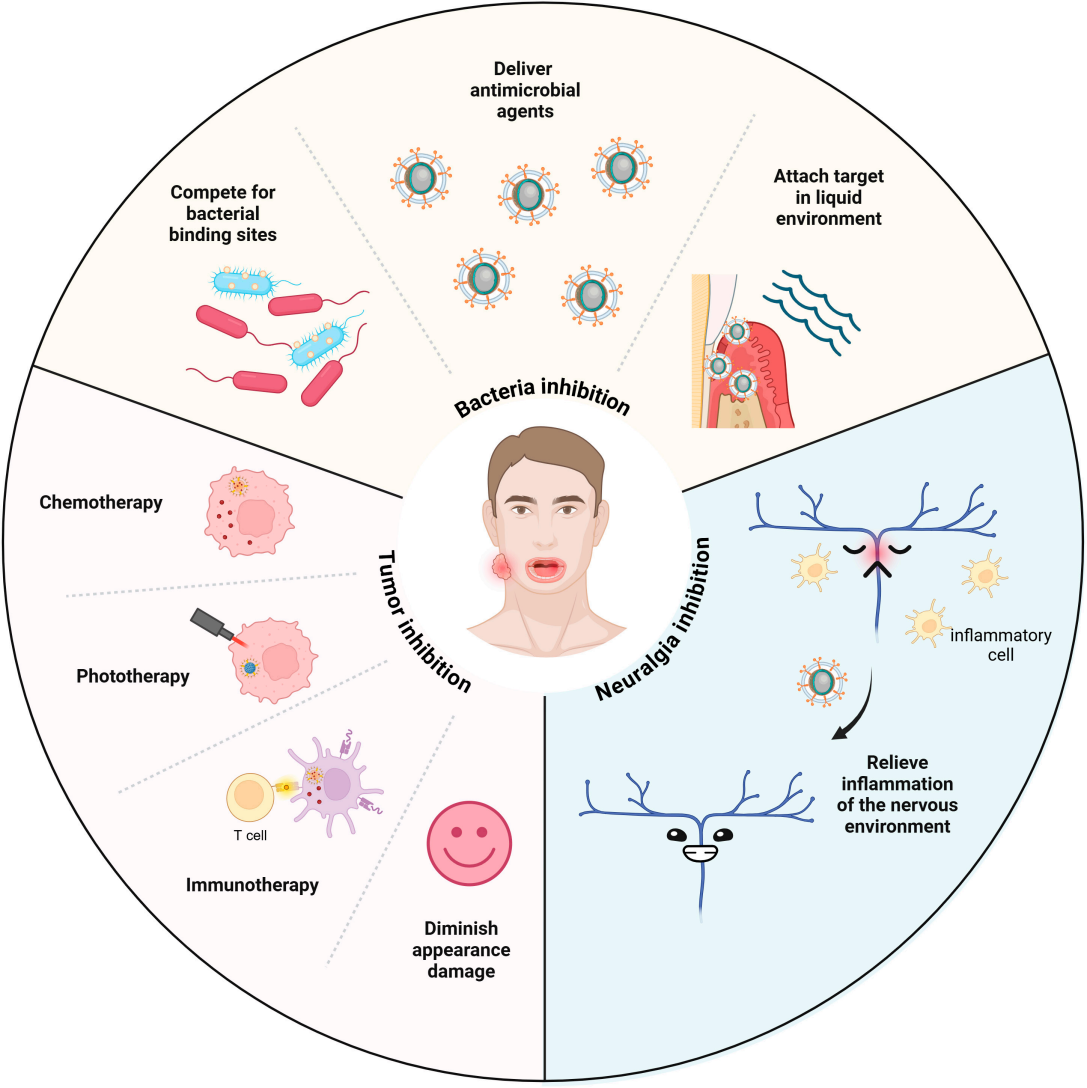
The importance of CMCNPs applied in dental, oral, and craniofacial region. The advantages of CMCNPs for dental, oral, and craniofacial diseases include bacteria, neuropathic pain, and tumor inhibition.

Combined with the characteristics of dental, oral, and craniofacial diseases like micro, multiple, and difficult to cure, CMCNPs will be excellent treatment tools, which can be summarized as inhibiting bacteria, neuropathic pain, and tumors (Fig. 1). The formation of dental plaque biofilm relies on bacterial adhesion, where cell membrane factors compete for attachment sites. Antibacterials delivered via this membrane target plaque biofilm, forming stable bonds to withstand oral fluids. Nerve growth factor (NGF) sustained release triggers neuropathic pain, yet selective reactive oxygen species (ROS) removal around M1 macrophages inhibits their polarization, decreasing NGFs and pain. CMCNPs, modified chemotherapy drugs, enhance efficacy in chemo-, photo-, and immunotherapy, offering a less invasive alternative to surgery while preserving patient aesthetics.

The imbalance of oral flora is closely related to dental caries, periodontitis, halitosis, and oral ulcers, while it is hard to prohibit the proliferation of particular bacteria. CMCNPs demonstrate their advantages in antibacterial quality. NPs coated with bacterial membranes can compete with parental bacteria for the binding site of the host, thus resisting the adhesion of parental bacteria [60]. The direct adhesion by bacterial surface protein or indirect adhesion by plasma-bridging molecules between PLTs and bacteria is beneficial for the targeted delivery of antibiotics, which is propitious to pass antibiotic resistance mechanisms [61]. For example, *Porphyromonas gingivalis* (*Pg*), an important pathogen of periodontitis, can specifically interact with FcγRIIa of PLTs through the surface adhesive Hgp44 on the membrane under the mediation of serum antibodies [62]. Another advantage for CMCNPs in dental and oral diseases is prolonging drug action time. Due to the erosion of a large amount of gingival crevicular fluid and saliva, and the movement of the tongue, it is difficult for traditional NPs to stay in the periodontal pocket for enough time to play a sufficient effect of drugs. The biocompatibility and targeting ability of CMCNPs make the drug act on the target stably and improve the drug utilization rate. In the present research, the antibacterial rate of CMCNPs and NPs was 45% and 35%, respectively [63]. In terms of inflammation resistance, the CMCNP group exhibits a notable reduction in the release of inflammatory factors, such as interleukin-17a (IL-17a) and tumor necrosis factor-α (TNF-α), with levels nearly halved compared to those observed in the NPs group [64,65]. From the observation of effect, the correlation index of periodontal status of the CMCNP group is obviously better than that of the NPs group. The distance from the cement-to-enamel junction to the alveolar bone crest of CMCNPs and NPs was 0.5 and 0.75 mm, respectively [64]. The bone volume and the number and thickness of trabeculae of the CMCNP group are about 1.5 times those of the NPs group [63].

Neuropathy attributed to pulpitis can cause electric shock-like pain, which is caused by inflammation stimulating the trigeminal nerve. A nanomaterial that encapsulated MnO_2_-NPs with a TrkA-overexpressed macrophage membrane shows excellent efficiency in binding various inflammatory factors and inhibiting neural progenitor cells, which prompts us to develop DDSs for nerve pain inhibitors in the dental, oral, and craniofacial region [66]. Moreover, CMCNPs for dental, oral, and craniofacial diseases have been widely utilized in HNC for diagnosis and treatment, which show their specific advantages like immune escape, targeting, and biocompatibility. Compared with surgency when treating cancer, the trauma caused by CMCNP therapy is minute, thereby satisfying the aesthetic requirements of patients. Besides, the efficiency of CMCNPs in treating HNC is better than NPs. The research has shown that the tumor volume of the CMCNP group could disappear almost completely [67,68], or it would not increase basically after inoculation with cancer cells [53,54,69]. Despite the initial larger tumor volume observed in the NPs group, this disparity tended to exacerbate over time. Furthermore, in terms of in vivo drug distribution, CMCNPs exhibited a higher concentration within tumors compared to NPs, with reduced accumulation in the liver and kidney [68].

## Fabrication of CMCNPs

The cell membrane is a phospholipid bimolecular functional layer with a bilateral mismatch, and it is attached to a variety of proteins to perform its functions [70]. When isolating the cell membrane, it is crucial to preserve these components as intact as possible. Different categories of cell membranes serve different functions. Erythrocyte membrane surface protein can help erythrocyte membrane-coated NPs to prolong systemic circulation time and increase the distribution of NPs in the tumor through EPR effect [71]. Macrophage membrane can guide NPs to the inflammatory site and prevent the internalization or being swallowed up by MPS [72]. Dendritic cell (DC) membrane can specifically activate T cells and achieve lymph node (LN) targeting [73]. CCMs can achieve immune escape and target the same type of tumor [74]. Severing as multipotent cells, MSC membranes can deliver drugs to the injury or inflammation sites and even promote neural differentiation [75].

**Fig. 2.**
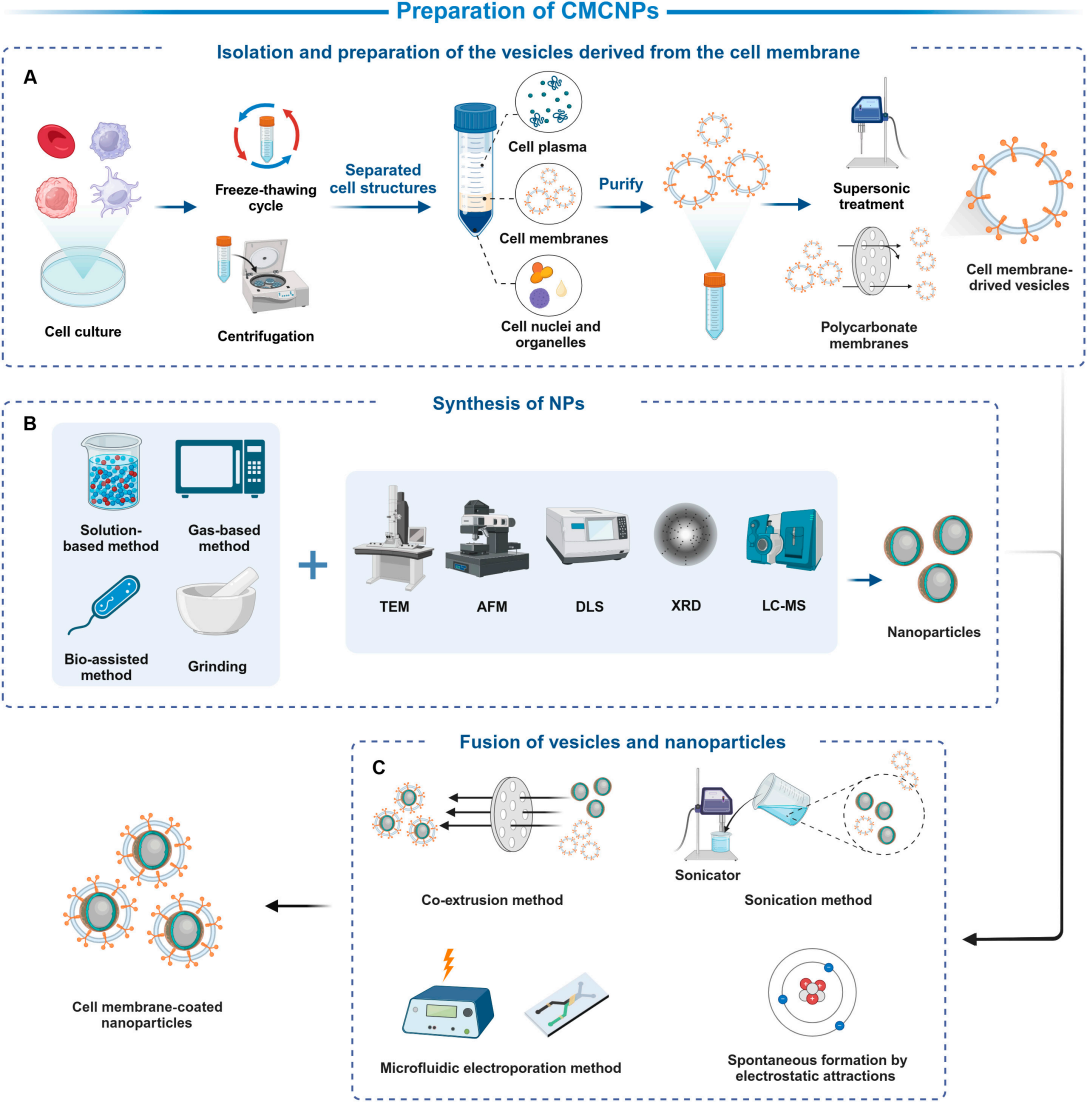
The fabrication of CMCNPs. (A) Acquisition of cell membrane vesicles from cell culture. (B) Manufacture techniques of NPs. (C) Fusion techniques of vesicles and NPs. AFM, atomic force microscopy; DLS, dynamic light scattering; LC-MS, liquid chromatography–mass spectrometry; TEM, transmission electron microscopy; XRD, x-ray diffraction.

The fabrication of CMCNPs includes 3 processes (Fig. 2): isolation and preparation of the vesicles derived from the cell membrane, synthesis of NPs, and fusion of vesicles with NPs [76]. The production of cell-mediated NPs offers the advantage of bypassing the intricate isolation and preparation processes associated with vesicles, thereby simplifying the overall manufacturing workflow.

### Isolation and preparation of the vesicles derived from the cell membrane

First, parent cells are selected and obtained according to the application purpose, and if they are scarce cells, they should be enriched in culture plates. Second, cytoplasmic components nuclei and organelles are removed through freeze-thawing cycles and centrifugation [77]. After that, the obtained pellet is repeatedly washed with phosphate-buffered saline (PBS) containing protease inhibitor until the supernatant becomes colorless [76]. After concentrating within a dispersion buffer, a pristine suspension comprising solely cell membranes can be isolated, ensuring high purity. Finally, cell membrane-derived vesicles with uniform size can be obtained after supersonic treatment and serial extrusion of the polycarbonate membranes [77].

### Synthesis of NPs

The techniques of NP synthesis involve the hydrothermal method, arc discharge method, chemical vapor deposition method, chemical oxidation method, ion sputtering method, laser pyrolysis, sol–gel process, electrodeposition, microemulsion method, and so on [78]. Biologic NPs can be simply divided into 2 categories: organic NPs and inorganic NPs. Organic NPs have better stability, such as lipid nanocarrier particles commonly used to transport nucleic acids and polymeric NPs that can be electrostatically combined with genes. Among organic materials, poly(lactic-co-glycolic acid) (PLGA) has proven great promise in clinical applications for its ability to prevent the formation of agglomerates when modified with various cell membranes [51]. Otherwise, a liposome is flexible enough to penetrate in vivo barriers but easy to decompose. Coated with a cell membrane, a liposome can achieve better stability and lengthen its circulation time [79]. Inorganic NPs have high specific surface area and strong surface plasma on resonance absorption but need to improve biodegradability [80]. Through the electrical, optical, and magnetic capabilities of inorganic NPs, CMCNPs can behave greatly in phototherapy [54,81]. At the same time, inorganic NPs coated by cell membranes can improve their biocompatibility. The choice of NPs materials should align with both the application requirements and the drug characteristics it delivers. Additionally, the size and shape of NPs should play crucial roles. NPs larger than 200 nm are more likely to accumulate in the liver and spleen, while NPs smaller than 5 nm are easily filtered out by the kidneys [82]. Discoidal NPs marginate to vessel walls more often than spherical NPs, possessing cracking margination dynamics [83]. The lateral drift velocity of particles is proportional to the aspect ratio of NPs [84]. Consequently, discoidal NPs exhibit superior drug protection and accumulation effects at targeted sites. What is more, the surface charge of NPs is related to protein adsorption, with high cationic NPs being more susceptible to removal from circulation compared to high anionic NPs [85]. However, it is neutral NPs and NPs with a slight negative charge that show notably prolonged cycle half-lives.

### Fusion of vesicles with NPs

The fusion of vesicles with NPs is the essential step in producing CMCNPs. Various techniques have been documented for these structures, encompassing co-extrusion methods, sonication techniques, microfluidic electroporation approaches, and the spontaneous formation facilitated by electrostatic attractions [76]. The most widely used method is the co-extrusion method, which can ensure that the size of CMCNPs is uniform and preserves the biological activity of the membranes [86,87]. This method is achieved by co-extruding NPs and vesicles through the gradually decreasing pore size of porous polycarbonate membranes, which, however, takes a lot of time and effort. Sonication method offers simplicity in its application, facilitating the reconstruction of the membrane around NPs in a single step [88]. However, it also carries the risk of causing irreversible damage to the membrane. Microfluidic electroporation method involves thoroughly mixing NPs and vesicles and enabling NPs to enter the transient pores that are generated on the membrane by electric pulses [89]. Besides, NPs and vesicles can spontaneously fuse through electrostatic attraction, offering another effective approach for membrane reconstruction [90]. This puts forward higher requirements for controlling the amount of charge on the surface of NPs.

Finally, the impurities in the CMCNP solution are removed by differential centrifugation or dialysis, and the purified CMCNPs are stored in the PBS (pH 7.4) solution containing a protective inhibitor below 4 °C [91].

## Applications of CMCNPs for Dental, Oral, and Craniofacial Diseases

**Fig. 3.**
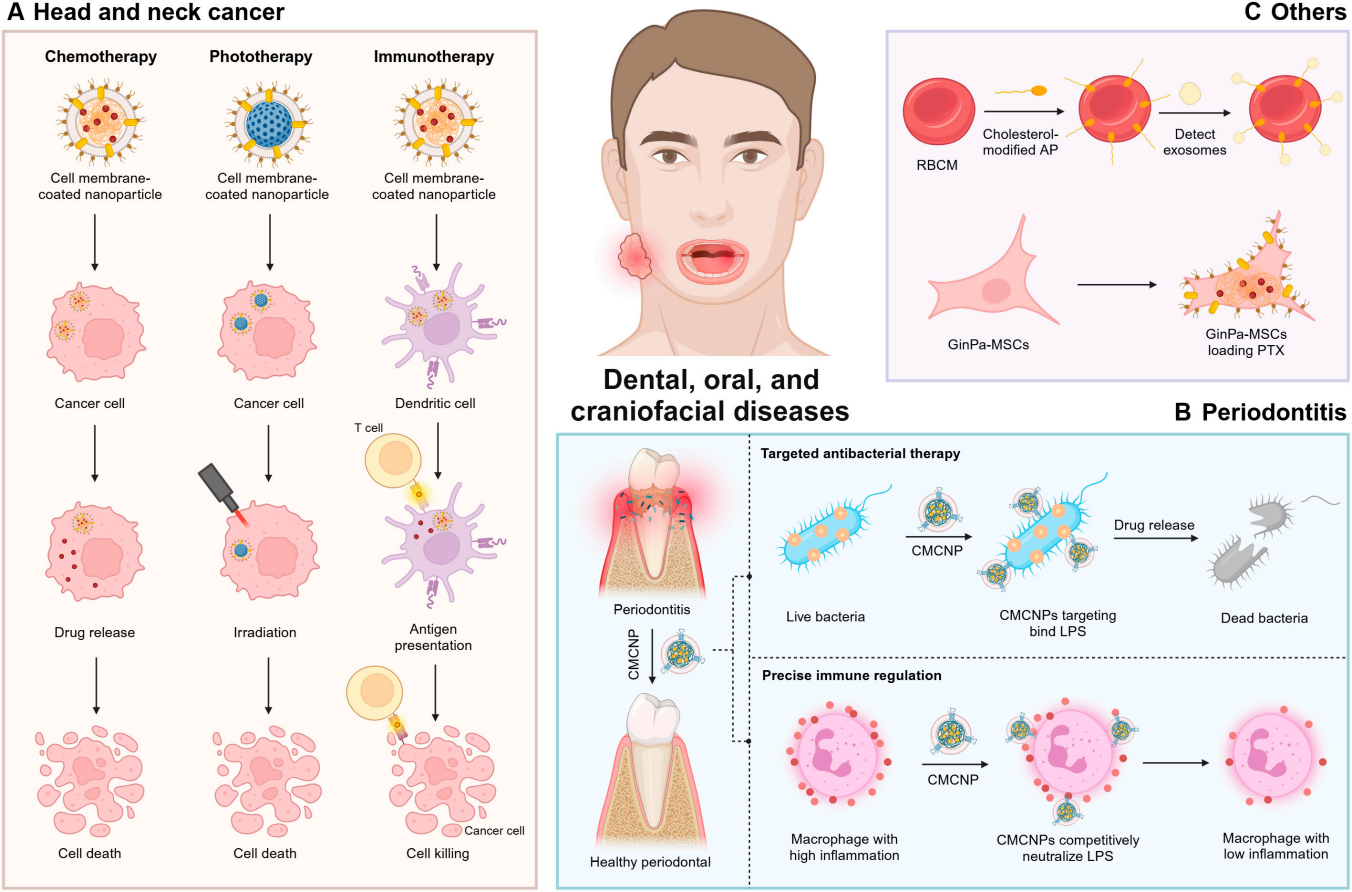
Applications of CMCNPs for dental, oral, and craniofacial regions. (A) CMCNP function process of chemotherapy, phototherapy, and immunotherapy for HNC treatment. (B) Application of a biomimetic NP manufactured from functioned cell membranes and NPs loaded with antibiotics with targeted antimicrobial and precise immune regulation through topical administration of periodontitis. (C) Other applications of CMCNPs for dental, oral, and craniofacial diseases (e.g., exosome detection and drug delivery). CMCNPs, cell membrane-coated NPs; GinPa-MSCs, gingival papilla mesenchymal stem cells; LPS, lipopolysaccharide; PTX, paclitaxel.

CMCNPs have shown great potential in drug delivery, particularly crucial for cancer treatment involving routine chemotherapy. Numerous researchers have applied CMCNPs for drug delivery in HNC, confirming their ability to prolong drug half-life, enhance targeting effect, and improve drug biocompatibility. In addition to their application in dental, oral, and craniofacial regions for the treatment of HNC, CMCNPs have also been utilized in the management of periodontitis, the detection of salivary exosomes, and the control of neuropathic pain (Fig. 3).

### Head and neck cancer

HNC is a cancer that develops in the mouth, nose, throat, salivary glands, and other head and neck areas, which includes nasopharyngeal cancer, oropharyngeal cancer, oral cancer, laryngeal cancer, hypopharyngeal cancer, and thyroid cancer [92]. It is a task to cure HNC due to its high recurrence and metastasis rates [93,94]. To improve the postoperative survival rate of HNC, surgeons will conduct radical resections of the lesion, irreversibly damaging the facial morphology and function of patients. In contrast to surgical interventions that can potentially compromise functions such as pronunciation and swallowing, CMCNPs utilized in DDS represent a substantially less invasive and more readily embraced therapeutic option.

#### Chemotherapy

Chemotherapy is a conventional treatment for cancers. However, due to issues like drug toxicity, drug clearance, and drug resistance, new requirements are put forward for targeting chemotherapy drugs. CMCNPs can solve these problems, as they show a great advantage in targeting tumor cells and low systemic toxicity. The cell membranes utilized for CMCNPs in HNC chemotherapy can be classified into 2 types: CCMs and MSC membranes. Cell adhesion molecules and intercellular molecules (especially lectins) highly expressed on the membrane surface of homologous cancer cells mediate the adhesion between tumor cells and contribute to the formation of tumor bodies in the progress of tumors [95]. Leveraging homologous targeting, chemotherapy NPs coated with CCMs precisely target tumors. MSCs’ CD47 marker enables immune evasion via Mas, while chemokine receptors like CXCR4, CXCR5, and CX3CR1 on MSC membranes steer CMCNPs toward tumor and inflammatory sites [34].

**Fig. 4.**
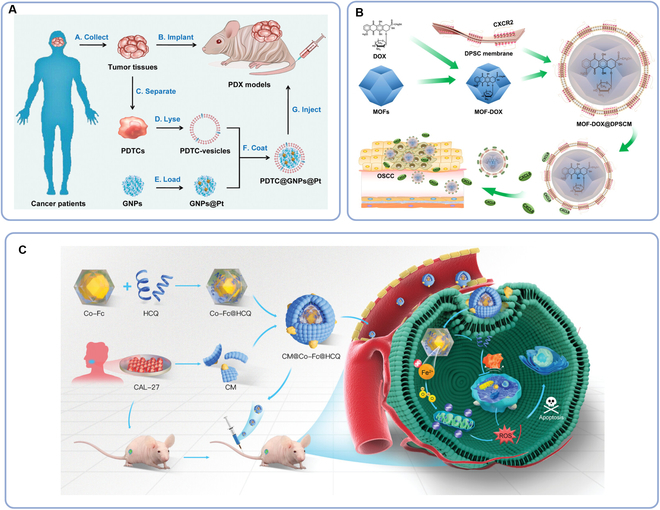
CMCNPs for chemotherapy in the treatment of HNCs. (A) Pt nanodelivery system mediated by PDTC [68]. (B) DPSC membrane modified with MOFs for DOX delivery [97]. (C) Nanoscale cobalt–ferrocene metal–organic framework incorporated with oral CCM for autophagy inhibitor HCQ delivery [69]. CM, cell membranes; Co–Fc, cobalt–ferrocene metal–organic framework; CXCL8, cryo-electron microscopy structures of IL-8; CXCR2, CXC chemokine receptor 2; DOX, doxorubicin; DPSC, dental pulp mesenchymal stem cell; GNPs, gelatin NPs; HCQ, hydroxychloroquine; MOFs, metal–organic framework NPs; OSCC, oral squamous cell carcinoma; PDTC, patient-derived tumor cell; PDX, patient-derived xenograft; Pt, cisplatin; ROS, reactive oxygen species.

Cisplatin (Pt) is a common anticancer drug, but the application of pure Pt will lead to severe weight loss. Rao et al. [68] used patient-derived tumor cell (PDTC) membranes to wrap gelatin NPs (GNPs) to deliver Pt, so a PDTC@GNP@PT treatment platform was constructed (Fig. 4A). The researchers constructed mouse tumor models by cancer cells isolated from HNSCC patients and proved that the homology PDTC@GNP@PT had advantages in anti-drug immune clearance rate, homology targeting, biocompatibility, and inhibition of tumor growth and recurrence. In addition, the results showed that the PDTC membrane did not interfere with the drug release process. PDTC@GNP@PT aids in tumor recurrence suppression, yet targeting efficacy diminishes in heterologous tumor models, likely due to factors like tumor site, malignancy, and individual variability. These complex mechanisms hold to be solved. Compared with other MSCs, gingival papilla MSCs (GinPa-MSCs) have better homology with oral cancer cells, which are suitably used in DDSs against oral squamous cell carcinoma (OSCC). Coccè et al. [96] compared the effects of GinPa-MSCs on releasing PTX, doxorubicin (DOX/DXR), and gemcitabine (GCB) in conditioned media (CM), and the release efficiency of GinPa-MSCs/DXR CM could achieve 100%. Furthermore, the secretoma of Ginpa-MSCs did not stimulate the in vitro growth of OSCC cells, whereas the drug-loaded GinPa-MSCs CM showed a substantial advantage in resisting tumor cell growth. However, when GinPa-MSCs were inoculated with anticancer drugs, the drugs would inhibit the growth of carrier cells, which needs to be solved in the future, so as to improve the fabrication efficiency of DDSs. CXCR2 distributed in dental pulp MSC (DPSC) is the receptor of CXC motif ligand 8 (CXCL8), a chemokine secreted by OSCC. Zhou et al. [97] modified metal–organic framework NPs (MOFs) with DPSC membranes (MOF@DPSCM), which could specificity target OSCC twice as much compared with unmodified MOFs (Fig. 4B). In vivo and in vitro experiments showed that MOF@DPSCM carrying DOX had obvious enrichment and killing effects on both CAT27 cell lines and OSCC tumors. Active autophagy of tumors can reduce stress reaction and accumulation of ROS [98], and weaken a reaction triggered by nanometal organic frameworks (NMOFs): Fenton reaction, which can generate ROS with the capacity of generating hydroxyl radical with strong oxidizing ability [99]. Therefore, Chen et al. [69] designed a nanoscale cobalt–ferrocene metal–organic framework incorporated with oral CCM to deliver the autophagy inhibitor hydroxychloroquine (HCQ) (CM@Co–Fc@HCQ) for OSCC treatment (Fig. 4C). CM@Co–Fc@HCQ utilized the synergistic effect of Co–Fc (produced ROS) and HCQ (inhibited autophagy) to enhance the killing effect of the tumor. However, this research was conducted on a subcutaneous tumor model rather than on an oral tumor model in situ or a human tumor xenotransplantation model.

Exosomes, secreted by cells, offer an alternative nanodrug delivery approach to HNSCC chemotherapy, bypassing cell membrane integrity concerns and simplifying production requirements [100].

#### Phototherapy

Phototherapy uses photosensitizer to absorb near-infrared (NIR) light (650 to 1,350 nm) to induce therapeutic responses, which has the advantages of minimally invasive and side effects [101]. Photothermal therapy (PTT) and photodynamic therapy (PDT) are 2 commonly used phototherapies: PTT is based on photothermal agent-mediated hyperthermia, while PDT is based on ROS released by photosensitizers [102]. PTT holds promise as a noninvasive therapy in cancer treatment, as it is not constrained by drug resistance [103]. However, the curative effect of single-use PTT or PDT is not satisfactory. Researchers found that PTT and PDT NPs can present antigens after being swallowed by DC and induce immunogenic cell death (ICD) to enhance the immune response [102,104]. Therefore, more and more research has focused on the combination of phototherapy and immunotherapy.

**Fig. 5.**
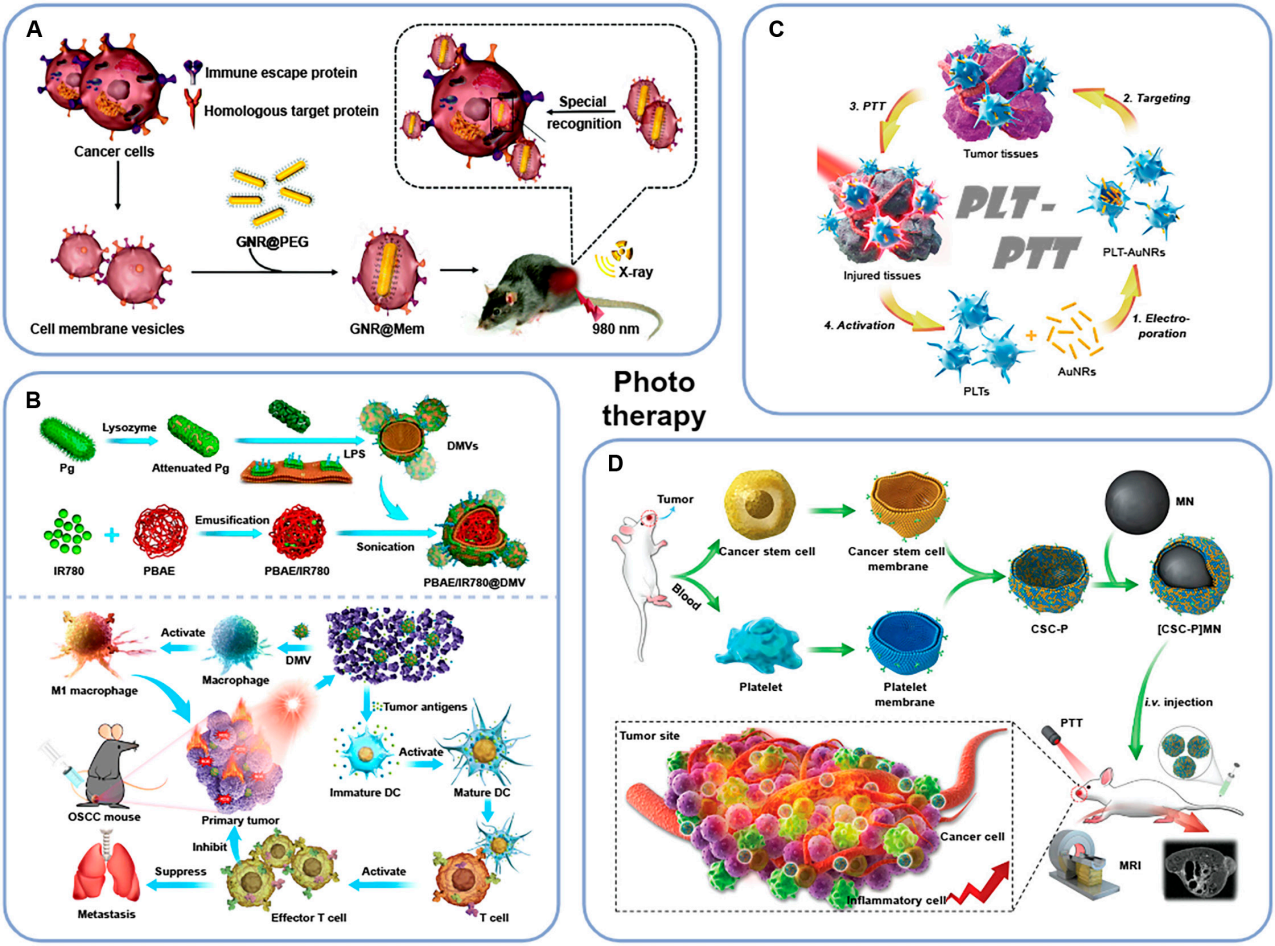
CMCNPs for phototherapy in the treatment of HNCs. (A) CCM-coated AuNRs for PTT and radiotherapy on OSCC [106]. (B) Biomimetic phototherapeutic nanoagent based on bacterial double-layered membrane vesicles for OSCC treatment [111]. (C) PLT-facilitated PTT of HNSCC [114]. (D) Cancer stem cell–PLT hybrid membrane-coated magnetic NPs for enhanced PTT of head and neck squamous cell carcinoma (HNSCC) [53]. [CSC-P]MN, CSC-PLT membrane-coated Fe_3_O_4_ NP; AuNRs, gold nanorods; CSC, cancer stem cell; DC, dendritic cell; DMVs, double-layered membrane vesicles; GNR, gold nanorod; GNR@Mem, CCM-coated gold nanorods; IR780, a near-infrared dye possessing both PDT and PTT capabilities; LPS, lipopolysaccharide; MN, iron oxide NP; MRI, magnetic resonance imaging; OSCC, oral squamous cell carcinoma; PBAE, poly(β-amino) ester; PEG, polyethylene glycol; *Pg*, *Porphyromonas gingivalis*; PLT, platelet; PTT, photothermal therapy.

In order to improve the accumulation of NPs in the tumor site when PTT therapy is applied, Wu et al. [105] made CCM-coated Au @ carbon core–shell NPs (Au@c-CCM). Proteins (e.g., carcinoembryonic antigen and galectin-3) on CCM can mediate adhesion to promote Au@c-CCM targeting tumor, and NPs lead to photothermal-mediated immunogenic death of tumor cells under NIR radiation. The experimental results of mice with tongue cancer model show that NPs can be fully accumulated in the experimental group compared with the control group, and it has an obvious inhibitory effect on tumor volume increase. A variety of preclinical transformation models were constructed, which can support the design and application of personalized biomaterials in different scenarios. However, Au@c-CCM can still improve its performance, such as improving the photothermal conversion efficiency of the Au core by adjusting the size or morphology. Similarly, Sun et al. [106] also used CCM to mediate HNSCC phototherapy (Fig. 5A). There were researchers using nano-photosensitizers coated with macrophage membranes to mediate PDT, resisting to oral cancer [107]. PTT can stimulate the production of ROS, thus triggering ICD [108,109]. To avoid immunosuppression of TME, bacterial adjuvant can be added to tumor vaccine to improve the antitumor immune response [110]. Chen et al. [111] derived the double-layer membrane vesicles (DMVs) from *Pg*, which were commonly found in the oral cavity, and encapsulated the poly (β-amino) ester (PBAE) NPs loaded with IR780 (a NIR dye with PDT and PTT ability) into the membrane (PBAE/IR780@DMV) (Fig. 5B). PBAE/IR780@DMV realized the synergistic therapeutic effect of PDT/PTT and immune stimulation on OSCC.

Compared with other cells in the TME, cancer stem cell (CSC) has been proven the hardest targeting one, which is the “killer” of cancer spread and recurrence [112]. By preparing CSC and PLT hybrid membrane (CSC-P) for DDS, NPs can achieve both the isotype targeting ability mediated by CSC adhesion molecule (CD44) and the immune escape function mediated by PLT membrane surface marker (CD47). Bu et al. [53] coated Fe_3_O_4_ magnetic NPs with CSC-Pm([CSC-P]MNs) and applied [CSC-P]MNs to PTT against HNSCC tumor (Fig. 5D). The heat energy generated during PTT treatment will damage tumor tissues, thus recruiting PLTs, the “sentinel” in blood circulation [113]. Rao et al. [114] loaded gold nanorods (AuNRs) into PLT (PLT-AuNRs) by electroporation (Fig. 5C). The tumor targeting of PLT promoted PTT in tumor tissue, and the tissue damage caused by PTT would recruit more PLT-AuNRs, forming a positive feedback process. In addition, the researchers found that compared with PLT membrane-camouflaged aunrs (PLT-m-aunrs), PLT-AuNRs had better immune evasion performance. This suggested that in order to improve the immune evasion performance of drugs, retaining as many components of carrier cells as possible could be taken into account.

#### Immunotherapy

Immunotherapy is to resist the immune escape of cancer cells by reactivating the antitumor immune response. Commonly applied immunotherapy includes restoring the function of T cells by combining immune checkpoint inhibitors with immune checkpoint proteins [including cytotoxic T lymphocyte antigen-4 and programmed cell death protein 1 (PD-1)] and modifying T cells in vitro to produce specific antitumor reactivity [115].

**Fig. 6.**
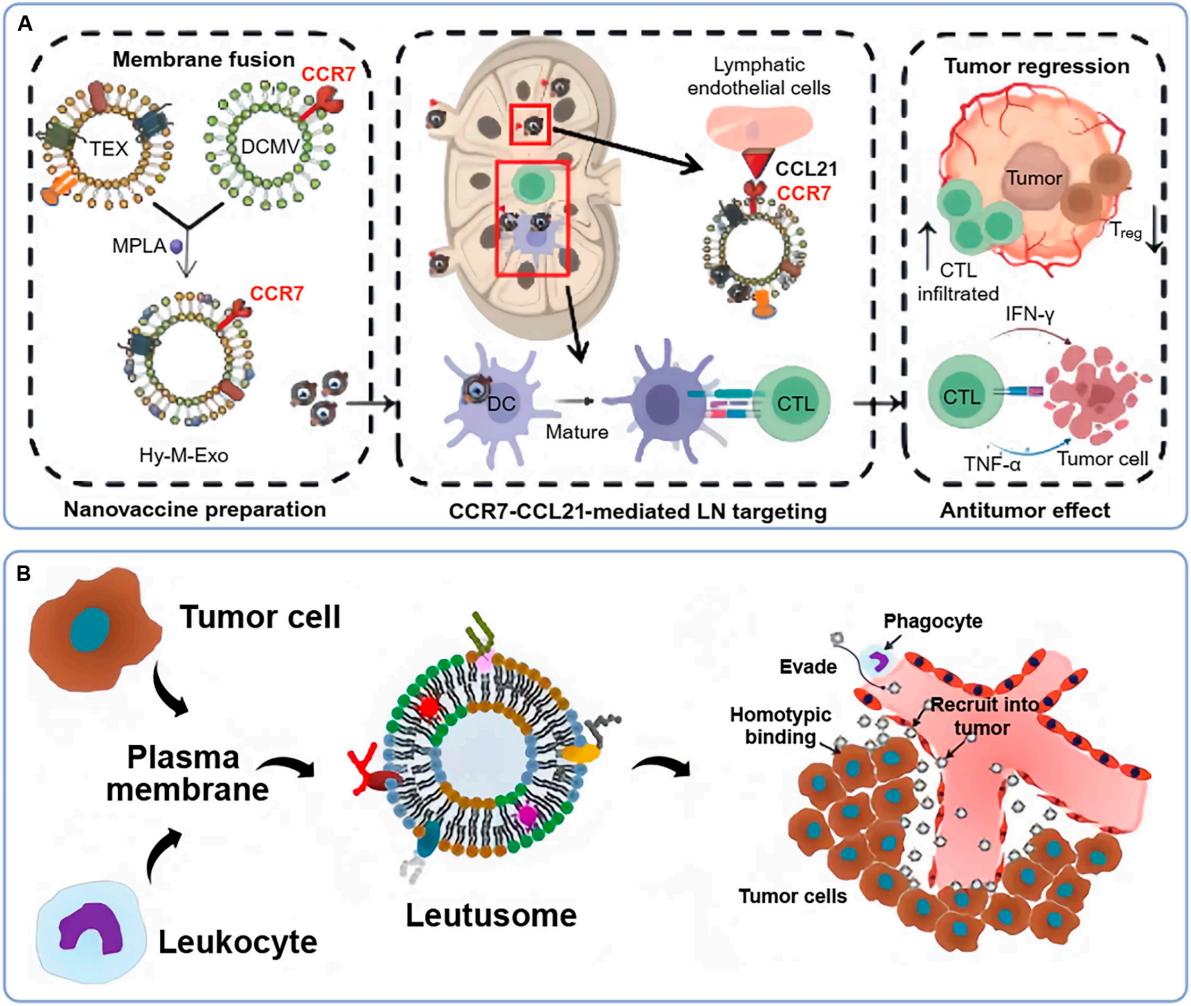
CMCNPs for immunotherapy in the treatment of HNCs. (A) Hybrid nanovaccine formed from TEX and CCR7 retained DCMV for HNSCC treatment [33]. (B) Biomimetic nanoplatform integrating plasma membrane components of leukocytes and tumor cells for remarkably enhanced solid tumor homing [54]. CCL21, CC-motif chemokine ligand 21; CCR7, CC-chemokine receptor 7; CTL, cytotoxic T lymphocyte; DC, dendritic cell; DCMV, dendritic cell membrane vesicle; Hy-M-Exo, a hybrid nanovaccine; IFN-γ, interferon-γ; MPLA, monophosphoryl lipid A; TEX, tumor-derived exosome; TNF-α, tumor necrosis factor-α.

Xu et al. [33] fused tumor-derived exosome and CC-chemokine receptor 7 (CCR7) retained DC membrane vesicle to develop a hybrid nanovaccine (Hy-M-Exo) for HNSCC therapy (Fig. 6A). Hy-M-Exo can deliver drugs to LNs through the CCR7-CCL21/19-mediated pathway and substantially activate DC and T cells, which can cope with HNSCC lacking defined tumor-associated antigens (TAAs). To improve the immune escape performance of CCM camouflaged biomimetic nanoplatforms, He et al. [54] designed a composite plasma membrane whose components are derived from both leukocytes and tumor cells, which is named leutusome (Fig. 6B). Leutusome has a good drug-carrying capacity for drugs with poor water solubility, so researchers use leutusome to deliver PTX and verify the immune escape performance and targeting of nanoplatform by fluorescence labeling. Compared with the control fluorescence signal in tumor cells, the half-life of NPs could be as long as 20.2 h. However, to optimize the immune response of leutusome, the membrane chimeric ratio between leukocytes and tumor cells has yet to be determined. Chen et al. [116] coated NPs carrying programmed death 1 small interfering RNA (PD-1 siRNA) with the tumor cell membrane (M-SNPs). The subcutaneous transplanted tumor model of SCC7 mice was treated with M-SNPs, and the expression of PD-1 in lymphocytes decreased substantially, thus relieving the inhibition of T cells and activating the immune response of tumors.

#### Combination therapy

The combination of above therapies can achieve the effect that is greater than the sum of its parts. Madsen et al. [117] used macrophages that internalize a toxic number of gold nanostars (Au NSs) for the effective delivery, thus realizing mild PTT (MPTT) treatment of a mouse model (the temperature that is higher than cytotoxicity induced by MPTT is 46 to 50 °C, while PTT is more than 50 °C). MPTT can enhance the efficacy of chemotherapy by increasing cell uptake, promoting endosome escape and drug release into the cytosol. DOX coated with HSC-3 CCMs (CC@DOXNPs) can be selectively delivered to OSCC. However, the drug release of CC@DOXNPs is lower than that of DOXNPs, which may be because the cell membrane is not conducive to the internalization rate of NPs. To improve the cancer inhibition efficiency, Li et al. [67] further modified the cell membrane with lip-indocyanine green (lip-CC@DOXNPs). Under NIR illumination, chemotherapy and PTT therapy can be combined, and the tumor growth inhibition rate is as high as 70.7%.

Dysfunctional tolerant DC and tumor-associated macrophages (TAMs) in the microenvironment of immunosuppressive tumors are the main reasons for immune tolerance [118]. Besides, the polycomb ring finger oncogene *BMI1* can promote metastasis and deterioration of HNSCC [119]. Wu et al. [120] utilized hydrogel to graft 2 types of NPs: CCM-coated mesoporous silica NPs (MSNs) containing paclitaxel (Pac) and a peptide-based proteolysis-targeting chimeras (peptide PROTAC, Pep) (PepM@PacC), and R837-loaded CaCO_3_ NPs (RC). PepM@PacC particles could be delivered to homologous cancer cells mediated by releasing PROTAC peptide to degrade *BMI1* and releasing Pac to induce cell apoptosis. At the same time, RC particles selectively manipulated TAM and DC to activate the T cell immune response. However, the dosages of administration for this nanodelivery platform has yet to be determined. This study was evaluated in a mouse cell-derived model, but it is difficult to reconstruct immune cells in humanized patient-derived xenografts engrafted with either human hematopoietic stem cells or human peripheral blood mononuclear cells.

LN metastasis is an important process in the occurrence and development of HNSCC. To inhibit the progress of HNSCC, it is vital to target circulating tumor cells (CTCs) in lymphatic vessels and LNs [121,122]. Li et al. [123] developed cancer membrane-coated digoxin (DIG) and DOX co-encapsulated PLGA NPs (CPDDs) that could target the homologous primary tumor cells and CTC clusters in lymphatic circulation. DIG as a Na/K adenosine triphosphatase inhibitor can produce negative impaction on the formation of tight junctions and desmosomes in CTCs and the epithelial–mesenchymal transition of the primary tumor [124]. With the homologous targeting by CCMs, CPDDs can target the CTC clusters in the lymphatic circulatory system and inhibit tumor metastasis. In lymphatic and CTC cluster metastasis models, the CPDD group showed advantages in both the size of LNs and the number of metastasis LNs. As an immune adjuvant, R837 can activate APCs [125] . To inhibit lymphatic metastasis, the diameter of CCM-coated NPs should be more minor than 200 nm. Li et al. [126] compared 3 different diameters of R837-loaded multi-antigen NPs MANPs/R837 coated with CCMs (MANP83/R837, MANP103/R837, and MANP22/R837). The mouse model showed that MANP83/R837 performed better immunostimulating ability than other experimental groups, and the combination with anti-PD-1 therapy remarkably inhibited tumor metastasis. Manganese oxide (MnOx) NPs exhibit Fenton-like activity in the TME and therefore induce the death of tumor cells, realizing chemodynamic therapy. Meanwhile, MnOx NPs can augment ICD to mediate the immune response. Li et al. [127] coated manganese oxide-loaded poly(2-diisopropylaminoethyl methacrylate) (MP) NPs with a hybrid cell membrane (RHM) derived from manganese oxide-remodeled 4T1 cells and DCs (MP@RHM). The homologous tumor-targeting molecules and chemokine receptors on RHM can drive LN homing and active DCs and T cells, and MnOx NPs induce cell death.

### Periodontal diseases

The coverage of patients with periodontal disease is extremely large, and the onset age includes children to the elderly [128]. The occurrence of periodontal disease involves the attachment of oral bacterial antigens and viral DNA/RNA and peptides to Toll-like receptors (TLRs) on immune cells, which will activate the immune response. However, the long-term effect of simultaneous inflammation will induce the imbalance of T cells and B cells, resulting in immune deficiency [5]. Studies have shown that periodontal disease and cancer are closely related, which is related to common risk factors and long-term inflammatory stimulation [129]. How to deal with various pathogens (e.g., bacteria and viruses) involved in periodontitis is the key to treating periodontitis [128].

**Fig. 7.**
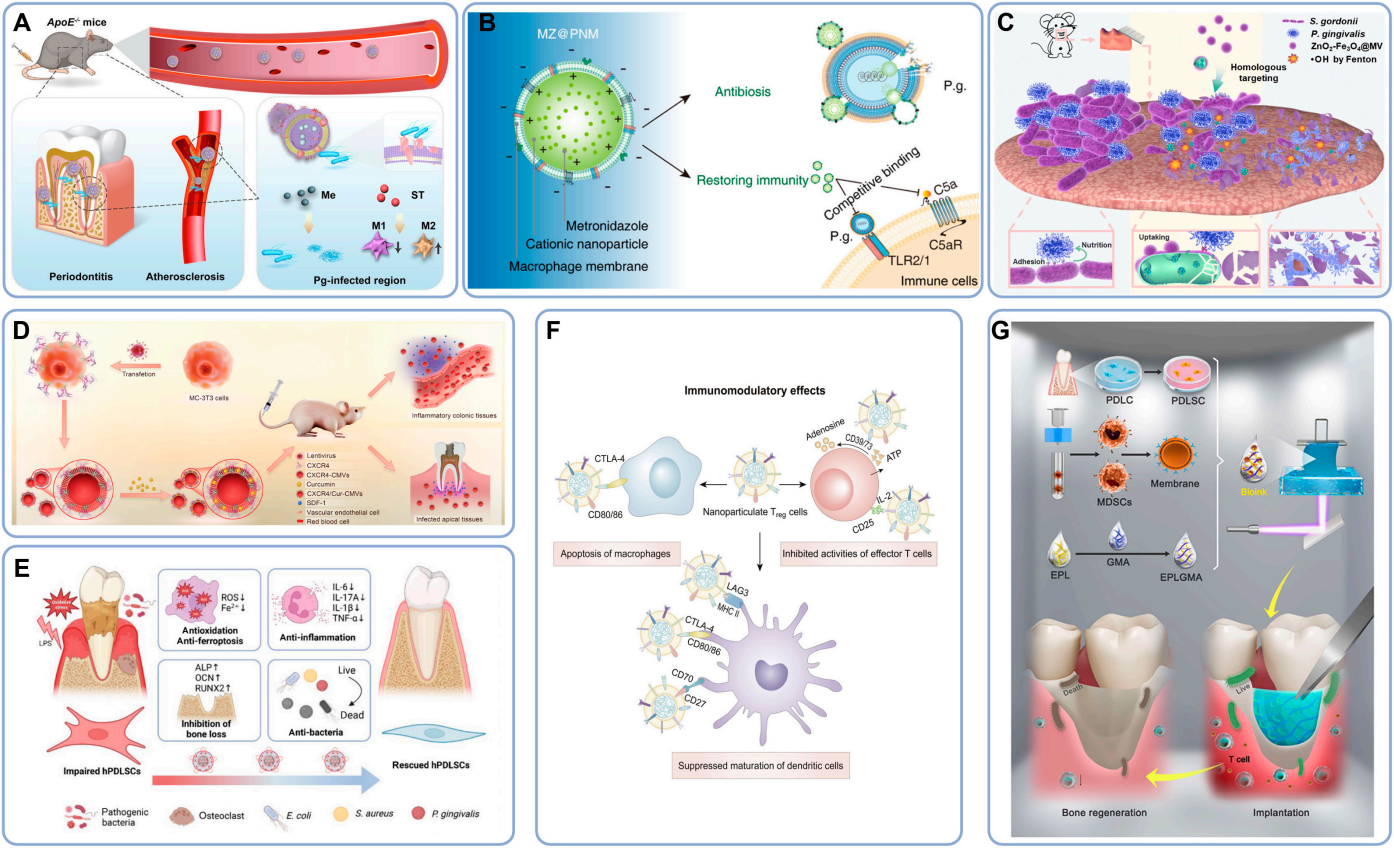
CMCNP treatments of periodontitis. (A) Macrophage membrane-coated nanodrug delivery system targeting *Pg* [131]. (B) Penetrating macrophage-based nanoformulation-encapsulating hydrogel targeting *Pg* [132]. (C) *S. gordonii* membrane-coated H_2_O_2_ self-supplied nanocomposite for *S. gordonii* inhibition [134]. (D) Cell membrane vesicles with enriched CXCR4 targeting inflammatory sites [138]. (E) Polydopamine NP based on gingival fibroblast membrane [65]. (F) Nanoparticulate regulatory T cells for inhibiting inflammatory reaction of periodontitis [64]. (G) 3D-printed bioink loading with stem cells and cellular vesicles for periodontitis-derived bone defect repair [63]. ALP, alkaline phosphatase; *ApoE^−/−^*, apolipoprotein E knockout; ATP, adenosine triphosphate; C5a, component 5a; C5aR, C5a receptor; CD, cluster of differentiation; CMVs, cell membrane vesicles; CTLA-4, cytotoxic T lymphocyte-associated protein-4; Cur, curcumin; CXCR4, CXC chemokine receptor 4; EPL, ε-poly-l-lysine; EPLGMA, GMA-modified EPL; GMA, glycidyl methacrylate; hPDLSCs, human periodontal ligament stem cells; IL, interleukin; LAG3, lymphocyte-activation gene 3; LPS, lipopolysaccharide; MDSCs, myeloid-derived suppressive cells; Me, metronidazole; MHC II, major histocompatibility complex class II; MZ@PNM, metronidazole @ penetrating nanomacrophage; OCN, osteocalcin; PDLC, periodontal ligament cell; PDLSC, periodontal ligament stem cell; *Pg*, *Porphyromonas gingivalis*; ROS, reactive oxygen species; RUNX2, runt-related transcription factor 2; SDF-1, stromal cell-derived factor-1; ST, simvastatin; TLR, Toll-like receptor; TNF-α, tumor necrosis factor-α; ZnO_2_/Fe_3_O_4_@MV NPs, *S. gordonii* membrane-coated ZnO_2_ NPs shell assembled with Fe_3_O_4_ NPs.

Drug treatment of periodontitis can start with inhibiting inflammation and killing pathogens. In the process of bacterial periodontitis, lipopolysaccharide (LPS) on the outer wall of Gram-negative bacteria is the key factor for immunoassay, and it is also the executioner of inflammation-mediated pyroptotic cell death [130]. Deng et al. [32] constructed a macrophage membrane that could express TLR4, and coated it on the surface of silk fibroin NPs (SNs) loaded with antibacterial agent minocycline hydrochloride (Mino) (MSNC). The nanodelivery system that was named MSNCs could target bacteria through TLR4 and perform immune regulation. The results of the mouse model of ligature-induced periodontitis treated with MSNCs showed that alveolar bone loss improved substantially. Similarly, Guo et al. [131] utilized *Pg* pretreated macrophage membrane to disguise the nanoparticle loading containing metronidazole (Me) and simvastatin (ST), which can transform M1-type macrophage into M2 phenotype to play a anti-inflammatory role, substantially inhibit *Pg* in vitro, and show good alveolar bone recovery in vivo experiments (Fig. 7A). The TLR2/1 and complement component 5a receptor (C5aR)-dependent signaling pathways can mediate the prohibition of phagocytosis and *Pg* killing from Ma; therefore, cell membrane cloaking derived from P.g.-specific lipopolysaccharide (P.g.-LPS) pretreated macrophages can express the high-level TLR2/1 complex to target *Pg* and enough C5aR to neutralize C5a. Cationic NPs loaded on negatively charged macrophage membranes can target *Pg* by the TLR2/1 complex and promote Me penetration by destroying bacterial membranes through positive charge (Fig. 7B) [132].

It can not only effectively resist inflammation by neutralizing complement and killing *Pg*, but also accurately target *Pg* to avoid hurting host cells. Another method is to inhibit the initial colonizing bacteria on dental plaque biofilm so as to “cut off” the secondary following-up colonizing bacteria mediated by adhesin. *Streptococcus gordonii* is the leader of following-up colonization of *Pg*, and the combination of them will enhance their resistance to antibiotics or unpleasant stimuli [133]. Cao et al. [134] designed *S. gordonii* membrane-coated ZnO_2_ NPs shell assembled with Fe_3_O_4_ NPs (ZnO_2_/Fe_3_O_4_@MV NPs) (Fig. 7C). ZnO_2_ NPs decompose to release H_2_O_2_ under a low pH environment in the oral cavity after meals, followed by bacteria killing mediated by the Fe_3_O_4_ NP-catalyzed Fenton reaction. In vivo and in vitro experiments showed that the *S. gordonii* membrane has a better targeting effect than liposome delivery.

To reduce the inflammatory reaction of periodontitis, anti-inflammatory drugs or immunosuppressive regulatory T (T_reg_) cells can be used to inhibit the function of T cells. Curcumin (Cur) can regulate the anti-inflammatory response mediated by signal transducer and activator of transcription 3 (STAT3)/Janus kinase 2 (JAK2) and VEGF pathways, and its reaction with bacterial surface proteins can directly harm bacteria [135,136]. Synthetic antioxidants play an important role in antioxidant activity, anti-inflammatory activity, and drug-carrying capacity and can slow down the destruction of alveolar bone by mediating ROS and anti-inflammatory factors [137]. Wang et al. [138] prepared cell membrane vessels (CMVs) rich in CXCR4 (CXCR4-CMVs) by lentivirus transfection, which tended to the inflammatory site through the CXCR12/CXCR4 axis (Fig. 7D). Then, they encapsulated Cur into CXCR4-CMVs (CXCR4/Cur-CMVs) by physical capture, and this DDS can promote the polarization of circular M2 macrophages better than free Cur. In the animal model of apical periodontitis, the aggregation of inflammatory cells and the secretion of IL-6, IL-1β, and TNF-α in the CXCR4/Cur-CMV group decreased substantially. Pan et al. [65] loaded minocycline onto antioxidant poly-dopamine (PDA) NPs (PM) and coated PM with cRGD-modified cell membrane (PM@RCM), which showed good antibacterial and anti-inflammatory effects and saved human periodontal ligament stem cells (PDLSCs) to achieve periodontal tissue reconstruction (Fig. 7E).

T_reg_ cells utilize their own inhibitory cytokines and functional ligands on the cell surface to interfere with the activation of immune cells and eliminate abnormal self-attack [139]. However, direct injection of T_reg_ cells into periodontitis is not effective, because T_reg_ cells will differentiate into T helper 17 (T_H_17) cells, which will promote bone resorption. T_reg_ cell membrane-coated NPs (TNPs) could suppress osteoclast differentiation induced by macrophage downstream, inhibit DC maturation through CD80 or CD86, and down-regulate the expression of CD4 T cells (Fig. 7F) [64]. In both murine and preclinical canine models of periodontitis, the TNP group exhibited a notable reduction in alveolar bone loss, demonstrating its efficacy in preserving periodontal health. To enhance the therapeutic efficiency and safety of TNPs, it still needs to integrate the drug payloads into the platform and ensure the dosages. The purification procedures and large-scale manufacturing of TNPs are also issues faced by clinical transformation.

Researchers have stored drugs, cells, and cell vesicles in a medium instead of directly encapsulating drugs in cell membranes to construct therapeutic modules. The antibacterial hydrogel ink based on glycidyl methacrylate (GMA)-modified ε-poly-l-lysine (EPL) (EPLGMA) served as a carrier, and the membrane proteins on the surface of myeloid suppressor cells (MDSCs) exerted anti-T cell and osteogenesis effects, while PDLSCs were loaded to promote bone regeneration (Fig. 7G) [63]. This 3-dimensional (3D) printing ink could adapt to various structural shapes. In vivo experiments showed the inflammatory regulation by MDSCs-MV, and CD4 and CD8 T cells decreased substantially. In osteogenesis, the experimental group is superior to the control group in bone regeneration and alveolar bone quality improvement, and the number of osteoclasts is reduced.

Various cells or cell membranes can be used according to the requirements to wrap nanotoxoid against bacteria to construct bacterial infection toxoid vaccines [140]. In particular, it is necessary to overcome the common bacteria in periodontitis such as *Pg*, przewalskii intermedia, and actinobacillus. We can also consider finding common targets of various bacteria and building one CMCNP platform to control these bacteria.

### Others

In addition to the above applications, the applications of CMCNPs in dental, oral, and craniofacial also include the detection of saliva contents and DDS mediated by CMCNPs from dental, oral, and craniofacial. The exosomes in saliva contain full-spectrum biomolecules derived from primitive cells, which can indicate diseases of the body [141,142]. Compared with blood tests, this saliva test is the “gold standard” for noninvasive detection. He et al. [143] established RBCs with a large number of CD63 aptamers on the surface and coated the electrode with RBCM obtained from these RBCs. There were a lot of AgNPs that were prepared and modified with a DNA probe SP (SP-AgNPs) on the surface of the electrode, which could bind to exosomes and produce an electrochemical response. When exosomes were captured by this nanoparticle platform through the CD64-mediated pathway, SP-AgNPs could respond and detect exosomes sensitively. Similar to inhibiting bacteria in periodontitis, CMCNPs were also used to treat oral mucosal diseases infected by fungi. Ye et al. [144] applied the extrusion method to encapsulate *Streptococcus salivarius* K12 (*S. salivarius* K12) membrane on triclosan (TCS)-loaded PLGA NPs (K12/TCS@PLGA-NPs), which had a drug encapsulation efficiency as high as 47.9% and drug-loading efficiency as high as 9.4%. Since *S. salivarius* K12 membrane can bind to *Candida albicans*, TCS@PLGA-NPs has a good adhesion rate to oral *Candida* and oral mucosa (67.2% adhesion rate of the oral mucosa; 68.87% adhesion rate of oral *Candida*) and realizes slow and sustained drug release. The excellent therapeutic effect of TCS@PLGA-NPs on oral candidiasis has been verified in mouse model.

In addition, researchers utilized GinPa-MSCs to deliver PTX against pancreatic carcinoma cells [31]. Compared with MSCs obtained from bone marrow, obtaining GinPa-MSCs is less invasive and these MSCs still perform well in drug loading and releasing.

## Challenges and Prospectives

Dental, oral, and craniofacial diseases are prevalent, posing life-disrupting challenges. CMCNPs address these administration hurdles, offering extended drug half-life, enhanced penetration, remarkable targeting, and immune evasion. Although the membrane may influence release, the cumulative effect of CMCNPs at the target surpasses nonmediated platforms [24]. Their fabrication, involving membrane vesicle isolation, NP synthesis, and vesicle–NP fusion, is simpler than modifying NP targets, vastly conserving resources [76].

When choosing the type of cell membranes or NP materials, it is necessary to assess their ease of integration with NPs. Besides, during the fabrication of CMCNPs, manufacturers should pay special attention to minimizing the damage to cell membranes caused by the manufacturing process as minor as possible. Furthermore, the size and shape of NPs also have meaningful impacts on therapeutic effectiveness. When fabricating cell-mediated NPs, researchers devise methods to mitigate the toxicity of drugs toward inoculated cells so as to improve the production efficiency. While autologous cell membranes may be utilized, their inconvenience for large-scale clinical applications necessitates the establishment of a cell membrane repository. However, the source of cell membrane repository used in uniform large-scale production should be included in the category of ethical considerations. The challenge lies in deriving a large number of cell membranes and obtaining high-purity NPs under aseptic conditions, putting forward higher demands on manufacture crafts, which indicate that a unified evaluation standard should be established. There are technologies that contribute to the realization of large-scale production, such as tangential flow filtration, which can efficiently concentrate exocrine vesicles, and microfluidic technology, which can effectively coat membranes at low cost [145,146]. CMCNPs must be devoid of any viruses and pyrogens, and it is crucial to eliminate the denaturation of membrane proteins to prevent any potential immune response triggered by endogenous antigens.

Currently, CMCNPs for dental, oral, and craniofacial diseases are mainly applied in the treatment of HNC and periodontal diseases. Researchers delivered photothermal agents or photosensitizers for phototherapy, chemotherapeutic drugs, and immunosuppressants by CMCNPs. The cell membrane-modified NPs can even collaborate with the delivered drug to achieve positive feedback on the antitumor effect. For HNC treatment, CMCNPs mostly utilize homologous CCMs, macrophage membranes, and MSC membranes. To realize the multi-function of the membrane, hybrid cell membrane can be produced by the co-extrusion method. In periodontal disease treatment, the primary focus is on weakening the inflammatory response of immune cells and enhancing the ability of DDS to target bacteria, viruses, and other therapeutic microorganisms. Biomarkers should be screened based on the types of infected pathogens, and cell membranes capable of specifically binding to the targets should be chosen accordingly to modify the NPs for antibacterial drug delivery. Researchers focus on *Pg* now, but this is not the only pathogen of periodontitis, and it is also a good angle to set “obstacles” in each process of oral plaque biofilm evolution. This domain requires the collaboration of pharmacologists, bioengineers, and periodontists to further advance. Obviously, there is a need to broaden the application of CMCNPs in dental, oral, and craniofacial regions. Exploring their use in managing the progression of endodontic and oral mucosal diseases, such as neuropathic pain control [147], and facilitating recovery after implantation are crucial areas of research. Cell membrane-mediated delivery of up-conversion NPs (UC NPs) has demonstrated its utility in tumor fluorescence imaging in vivo [89,148,149]. Applying this technology to the early detection and investigation of cancer metastasis for dental, oral, and craniofacial cancers can enhance diagnostic accuracy and contribute to the development of more effective treatment plans. Further, combined fluorescence imaging with artificial intelligence can assist in diagnosing the progress of a tumor. The combination of intelligent flexible electronics and CMCNPs can even achieve point-to-point monitoring and automatic drug administration.

The technique of CMCNPs harnesses complex biological functions that are otherwise extremely difficult to synthesize and replicate. However, the clinical application of this technology faces a great challenge: high price. Patients who have undergone CMCNPs need to bear expenses amounting to tens of thousands of dollars, yet the survival extension is limited to mere months [150]. Efforts must be made to reduce costs by optimizing materials and manufacturing techniques, ensuring that this therapeutic approach does not impose an undue financial burden on patients. One potential approach is to choose heterogeneous biological cells/cell membranes that are more accessible and possess comparable functionalities. Such heterogeneous cells such as RBCs, PLTs, and primary immune cells can be obtained from suppliers of blood banks. For CMCNPs that are derived from homologous CCM preparation, a large bioreactor can be utilized for cell volume proliferation [151]. Besides, there are only preclinical trials rather than clinical trials on CMCNPs that have been conducted at the present stage. In preclinical trials, compared with the PBS group, the weight of the experimental group mouse model decreased slightly, with no statistical significance [33,53,68,69,114,116]. After the drug administration, the nude mice were killed, and the heart, liver, spleen, kidney, and other organs and blood of each group of nude mice were taken out for hematoxylin and eosin staining and blood biochemical detection, which showed that the organ morphology of each group of nude mice was not damaged [33,53,54,67,116,132,138]. In addition, in in vitro experiments, no abnormal hemolysis occurred, nor did it inhibit the viability of normal cells [33,65,67,105,120,132]. Although CMCNPs show excellent biosafety and few side effects in preclinical models, the discrepancies in drug responses to the model between human-derived cells and cell lines or animal models result in dubious efficiency. Patient-derived primary cells are difficult to obtain as tissues removed during biopsy will be discarded. The preparation and storage make it hard to carry out clinical trials. Additionally, before launching clinical trials, it is necessary to evaluate the biosafety of CMCNPs in vivo for a long period and prevent immunological rejection. After all, it is necessary to enhance the effectiveness of CMCNPs for DDSs and carry out large-scale clinical trials to verify their effects [23]. At present, the clinical transformation has a long way to go, and the strategy formulation of clinical supervision needs to be comprehensively considered.

## Conclusion

**Fig. 8.**
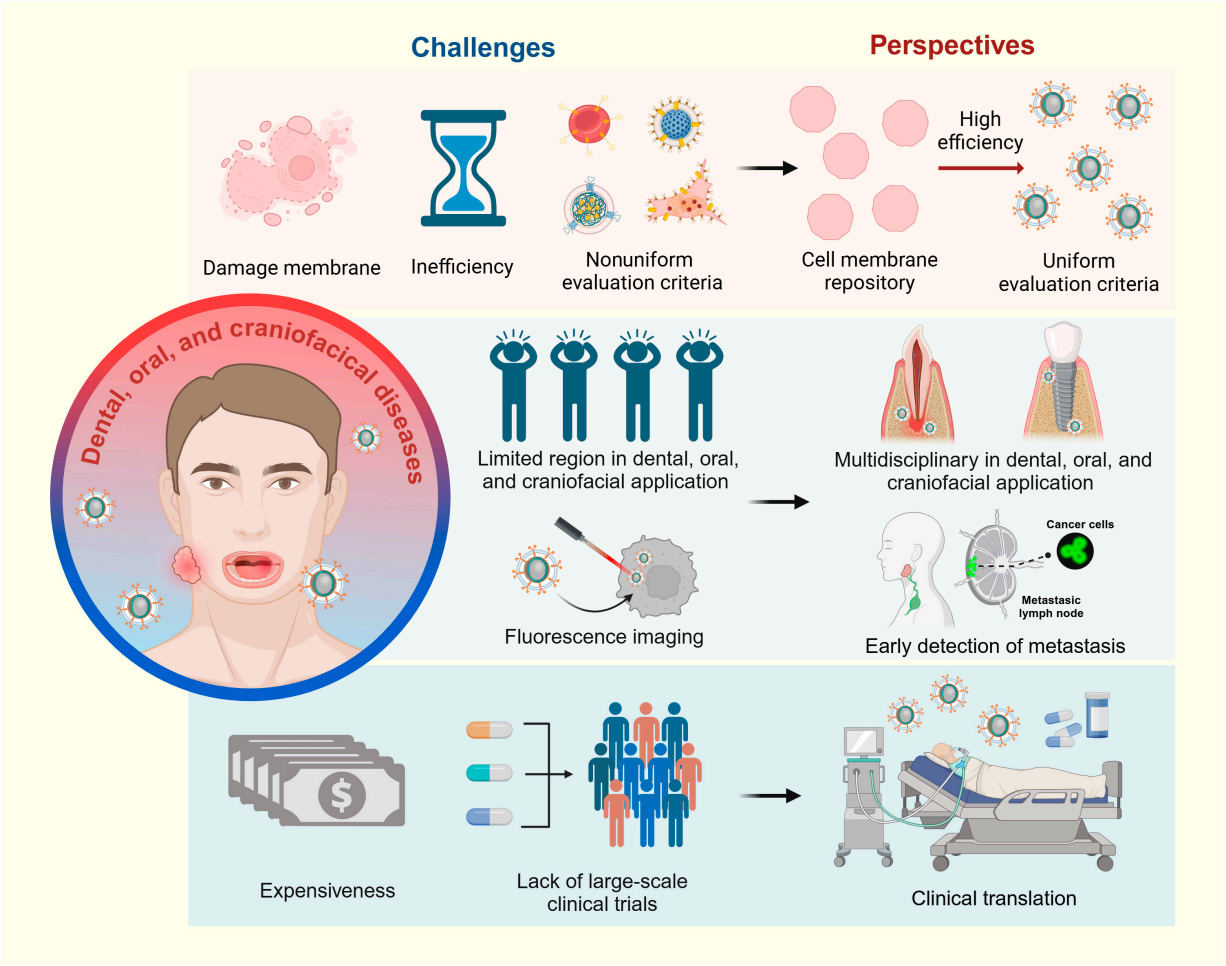
Future application directions for CMCNPs. CMCNPs for dental, oral, and craniofacial diseases should improve in 3 aspects: standardized and efficient production, expansion applying regions (e.g., endodontics and implant dentistry), and new techniques for early diagnosis, low pieces, and large-scale clinical trials for the guarantee of clinical application.

In summary, CMCNPs for dental, oral, and craniofacial diseases show advantages in chemotherapy, phototherapy, immunotherapy, and multi-method combined therapy, especially in prolonging the half-life of drugs and improving drug targeting. However, further efforts are required to translate this emerging technology into clinical application (Fig. 8). As this technology matures, it holds the potential to substantially contribute to enhancing human dental, oral, and craniofacial health.
